# Compressed-sensing-based fluorescence molecular tomographic image reconstruction with grouped sources

**DOI:** 10.1186/1475-925X-13-119

**Published:** 2014-08-20

**Authors:** Wei Zou, Xinyu Pan

**Affiliations:** School of Electronic and Information Engineering, Soochow University, Suzhou, 215006 China; Department of Electronic and Information Engineering, Hong Kong Polytechnic University, Hong Kong, China; School of Information Technologies, The University of Sydney, Sydney, NSW 2006 Australia; School of Electronics & Information Engineering, Suzhou University of Science and Technology, Suzhou, 215009 China

**Keywords:** Fluorescence molecular tomography, Inverse problem, Grouped sources

## Abstract

**Background:**

Although the quality of reconstructed results can be improved with the increment of the number of measurements, the scale of the matrices involved in the reconstruction of fluorescence molecular tomography (FMT) will become larger, which leads to the poor efficiency of the process of tomographic image reconstruction. In this paper, we proposed a new method for image reconstruction of FMT based on compressed sensing, in which a scheme of grouped sources is incorporated.

**Methods:**

The forward equations are implemented using the finite element method (FEM). The reconstruction model is formulated under the framework of compressed sensing theory. The regularization term and the total variation penalty are incorporated in the objective function. During the reconstruction of FMT, the sources are divided into two groups for iteration in turn. One group of sources is employed in the first iteration of inverse problem, and the other group is employed in the next iteration.

**Results:**

Simulation results demonstrate that the computation time and mean square error (MSE) of the reconstruction with our algorithm are less than those with the traditional method. The proposed algorithm can reconstruct the target with enhanced contrast and more accurate shape.

**Conclusions:**

The proposed algorithm can significantly improve the speed and accuracy of the reconstruction of FMT. Furthermore, our compressed-sensing-based method can reduce the number of measurements.

## Background

Optical molecular imaging has important applications in many fields such as molecular biology and clinical diagnosis, where disease-specific tracers are combined with the optical methods to detect and localize the abnormalities at their molecular stage [1]. Optical molecular imaging techniques further impart the potential ability to prevent and treat the lethal diseases. As one of the emerging optical molecular imaging techniques, fluorescence molecular tomography (FMT) plays a significant role and attracts considerable new interest due to its high sensitivity, low cost, and high-throughput capability [2]. FMT has been developed as a tomographic method to yield a robust modality for fluorescent reporters [3, 4]. FMT can be utilized in the studies of drug discovery, tumor diagnosis and therapy assessment. In this imaging modality, an external source of excitation light is needed to irradiate the tissues and then the injected fluorescent markers absorbs the incident light [5, 6]. Upon releasing the energy, the fluorophore emits the light at a longer wavelength and the fluorescent molecule decays to its ground state. The emission light is measured by measurement devices at the surface of the tissue. Monitoring the fluorescent markers is able to collect a large amount of functional information. The images of the absorption coefficient, fluorescent lifetime and the fluorescent yield can be reconstructed from the measured data and a mathematical model of light propagation [7].

Two processes are required to reconstruct the image of FMT. First, a forward model is utilized to map the parameters to the measurable data. Second, an inverse problem is used to calculate the spatial distribution of the optical and fluorescent properties when the measured data and sources are given. Ntziachristos *et al*. presented a normalized Born approximation to reconstruct the distribution of fluorochromes with different concentrations embedded in the media [8]. In image reconstruction of FMT, the quality of reconstructed results can be improved with the increasing number of measurement data. However, the scale of the matrices involved in the reconstruction will become larger, which may slow down the process of solving the tomographic inverse problem. Therefore, a new method based on compressed sensing (CS) is proposed in this paper to tackle such a problem and accelerate the reconstruction process. CS is an innovative information theory, which is able to recover sparse signals with the under-sampled measurements [9, 10]. Therefore, the CS-based method of FMT is capable of reconstructing the tomographic images with high speed and low cost from less number of measurements. This will provide potential benefit for biomedical applications. Besides the measurements, source is another important factor to determine the efficiency of the reconstruction. Since the fluorescence molecule is excited to emit the fluorescence light by the source, the position and number of sources can greatly affect the reconstructed results. The quality of reconstruction can be improved with the increased number of sources, which may lead to larger scale of matrix and higher computational requirements involved in the reconstruction process [11]. In [12], a model-order reduction approach is proposed for reduction of the system complexity. However, the transformation matrix is needed to be constructed with the Wilson–Yuan–Dickens basis vectors or the Lanczos basis vectors in the Krylov subspace. Therefore, we propose to implement the CS-based reconstruction with the grouped sources, which can improve the efficiency of the reconstruction process. Simulation results demonstrate that our proposed method can significantly speed up the reconstruction process with high reconstruction accuracy.

## Methods

### Forward model

In general, light propagation in the near infrared spectral window is well modelled by the radiative transfer equation (RTE). In order to reduce the computational complexity, the diffusion equation is employed instead. The diffusion equation is the simplest nontrivial approximation that results from the *P*_1_ approximation to RTE [13]. The diffusion equation is widely utilized in the modelling of the light propagation in tissue. Mathematically, the following diffusion equation depicts the process of excitation light propagation in the frequency domain [14].
1

In equation (1), the subscript *x* denotes the properties at the excitation wavelength; *D*_*x*_ (**r**) and *k*_*x*_ (**r**,*ω*) represent the optical diffusion coefficient and decay coefficient, respectively; Φ_*x*_(**r**, *ω*) and *Q*_*x*_ (**r**,*ω*) denote the photon density and excitation light source, respectively; ∇ represents the gradient operator.

The other diffusion equation, which depicts the generation and propagation of fluorescent light, is of the form:
2

In equation (2), the subscript *m* denotes the parameters at the emission wavelength; ; Φ_*m*_ (**r**,*ω*) represents the photon density; Other quantities that are included in equation (2) are the fluorescence lifetime *τ*(**r**), the fluorescence quantum efficiency *ϕ*, the diffusion coefficients *D*_*m*_ (**r**), and the decay coefficients *k*_*m*_ (**r**,*ω*). Further, *k*_*x*,*m*_ (**r**,*ω*) and *D*_*x*,*m*_ (**r**) can be obtained below:
34

where *μ*_*ax*,*mf*_ (**r**) are the absorption coefficients due to fluorophore; *μ*_*ax*,*mi*_ (**r**) are the absorption coefficients due to nonfluorescing chromophore;  are the isotropic scattering coefficients, and *c*_*n*_ = *c*/*n* represents the velocity of light in tissue.

The numerical solutions for the excitation and emission density distributions in equations (1) and (2) are obtained using the Robin type boundary conditions, which take the form as follows:
5

where **n**(**r**) is outward facing normal vector for the boundary ∂Ω, *b*_*x*,*m*_ (**r**) are the Robin boundary coefficients at excitation and emission wavelengths, which depend on the optical refractive index mismatch at the boundary [15].

The forward equations can be computationally implemented using the analytical methods or the finite element method (FEM). Basically, analytical methods may lead to inaccurate results due to simplified assumptions of properties or geometry [16]. The most significant superiority of FEM is versatility. Thus FEM is applicable to inhomogeneous property distributions and complex geometries. Herein we solve the forward equations with FEM. To facilitate a finite element solution of the forward equations, the reconstruction domain Ω is discretized into *P* elements with *N* vertex nodes. By means of the basis functions *ψ*_*i*_ (i = 1,2,…,*N*), the solution Φ_*x*,*m*_ can be represented by  [17]. Hence, making use of finite element discretization of equations (1) and (2), we can yield the matrix equation as shown below:
67

### Inverse problem

The inverse problem is to recover the distribution of tissue parameters *x* with a series of boundary measurements *y* as well as several sources. Let us denote the forward operator by *F*. Thus, the inverse problem can be represented by:
8

According to the Taylor series expansion [18], the linear inverse problem can be expressed by the following matrix form:
9

where Δ**x** denotes the changes in the optical properties, Δ**y** denotes the residual data between the predicted and measured data, and **J** denotes the Jacobian matrix.

### Compressed sensing

One important condition of CS is sparsity, which means that either the signal itself or its representation in an appropriate basis is sparse or compressible [19]. A discrete signal **x** ∈ ℝ^*N*^ is said to be *r*-sparse, if **x** contains *r* nonzero entries (*r* < <*N*). A discrete signal **x** ∈ ℝ^*N*^ is said to be *r*-compressible, if **x** can be represented by *r* large coefficients and other coefficients are small in magnitude. Basically, most medical images have sparse representations in some orthonormal basis (e.g., wavelet, Fourier). That is:
10

where **x** is the original image vector, **θ** is the transformed vector, and **Ψ** denotes the orthonormal basis.

A central idea in the CS theory is to acquire the signal **x** with the projection measurement vector **y** ∈ ℝ^*M*^ as follows:
11

where **Φ** is a measurement matrix with a size of *M* × *N*.

Substituting equation (10) into equation (11), we have:
12

According to the CS theory, the sparse solution **θ** in equation (12) can be obtained by solving the constrained optimization problem as follows:

min ||**θ**||_1_ subject to:
13

where ||||_1_ denotes *l*1-norm.

The unknown signal **x** can be ultimately recovered by equation (10).

### Compressed-sensing-based image reconstruction of FMT with grouped sources

For the linearized reconstruction, the perturbation from a homogeneous background or reference medium is relatively small in volume [20]. Therefore, sparsity can be pre-assumed for the linearized tomographic imaging problem. Typically in biological media, sparsity may be true if a very good reference medium is available. Thus, a transformation basis such as the Fourier basis can be utilized to reinforce the sparsity of the solution to equation (9) (i.e., Δ**x**) as follows
14

Based on the discussion in the above two sections, the problem of image reconstruction of FMT under the framework of CS theory can be expressed by:
15

According to equation (13), the sparse solution  in equation (15) can be solved by:

min  s.t.:
16

To implement CS-based reconstruction of FMT, the reconstruction model is formulated by:
17

where *E* is the objective function, ||||_2_ denotes *l*2-norm, *λ* denotes the regularization parameter [21], and *α* represents the parameter determining the sparsity. Basically, it will be useful to incorporate a total variation penalty into equation (17), which can be solved via the nonlinear conjugate-gradient method [22]. After obtaining the sparse solution , we can recover the solution Δ**x** in equation (9) by the transform as equation (14).

**Figure 1:**
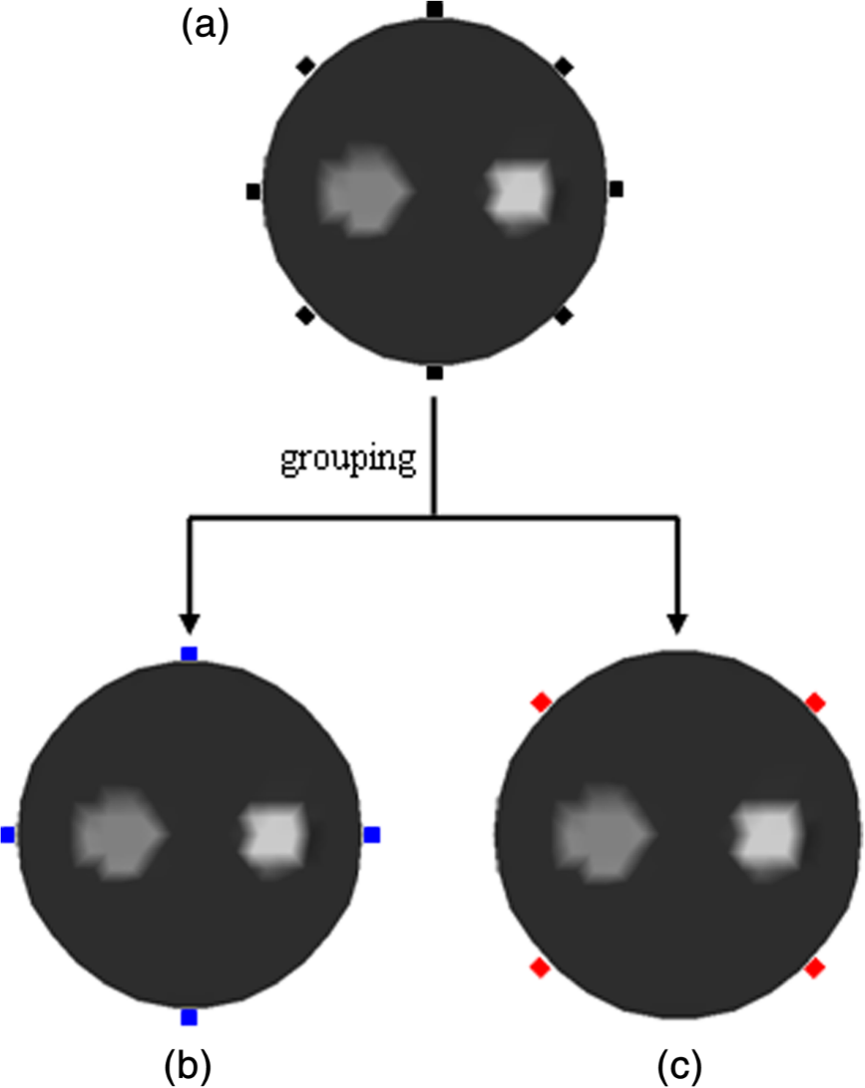
**Schematic of the iteration reconstruction based on the grouped sources. (a)** Sources without grouping, **(b)** sources of blue group, and **(c)** sources of red group.

As mentioned before, the measurements are generated with the excitation source for FMT. Therefore, the source placed in different position can provide different information to reconstruction. Furthermore, the number of sources can also affect the reconstruction results. More number of sources will improve quality of reconstruction, whereas the scale of the Jacobian matrix will increase. Consequently, the computational burden of the whole reconstruction may increase. Thus, in order to tackle such a problem, a scheme of grouped sources is proposed to improve a basic CS-based reconstruction of FMT. In this scheme, the sources are divided into two groups. One group of sources is employed in the first iteration of inverse problem, and the other group is employed in the next iteration. Two groups of sources can provide more information for image reconstruction than only one group of sources, which may improve the quality of reconstruction. More importantly, in this way, the number of rows in the Jacobian matrix can be reduced to only one half of that in the Jacobian matrix with all sources. Suppose the number of vertex nodes, sources, and measurements are *N*, *S*, and *M*, respectively. The computational complexity of solving the forward equations of FMT will be O (*N*^3^) + O (*N*^3^). Basically, the direct calculation of the Jacobian matrix with all sources needs *S* × (*N* + 1) FEM forward calculations. With the scheme of grouped sources, the calculation of the Jacobian matrix is reduced to only  FEM forward calculations. Thus, the computational requirements for the Jacobian matrix can obviously be reduced using the proposed algorithm. This is helpful for reducing the computational burden of the tomographic inverse problem. In addition, the iterative reconstructed results from one group of sources will offer a good initial guess for the next iteration of reconstruction from the other group of sources. Figure 1 shows the illustration of the grouped sources strategy. In Figure 1(a), the sources without being grouped are illustrated with black rectangles. Figure 1(b) and (c) display the two groups of sources with blue rectangles and red rectangles, respectively.

In fact, the solution to the inverse problem can be achieved by minimizing the following objective function:
18

where *J* (**x**) denotes the objective function measuring the discrepancy between the measured data **y** and predicted data *F*(**x**) with regards to the forward model. Therefore, the overall reconstruction algorithm consists of following steps

1. Set **x** = **x**_0_ with **x**_0_ being an initial guess;

Set *m* = 0;

(2) if (*m*%2==0) then

Compute Δ**y** and **J** at **x** with the first group of sources;

else

Compute Δ**y** and **J** at **x** with the second group of sources;

end if

Set *m* = *m* + 1;

(3) Solve equation (17) with the nonlinear conjugate-gradient method;

Recover Δ**x** using equation (14);

(4) Update **x** with **x** = **x** + Δ**x**;

Calculate the corresponding objective function *J* (**x**) by equation (18);

(5) if (*J*(**x**) < ***δ***) then

Stop and output **x**;

else

Return to step (2);

end if

## Results and discussion

In this section, two simulation experiments were performed to test the efficacy of the proposed algorithm: image reconstruction of a single fluorescence target, and two closely spaced fluorescence targets. The synthetic measurement data are generated from the diffusion equations as equations (1) and (2). In addition, random Gaussian noise with a signal-to-noise ratio of 10 dB is applied to these synthetic measurement data to simulate measurement error. The initial guess **x**_0_ in this study is set to 5 *mm*^-1^.

### Single fluorescent target

**Figure 2:**
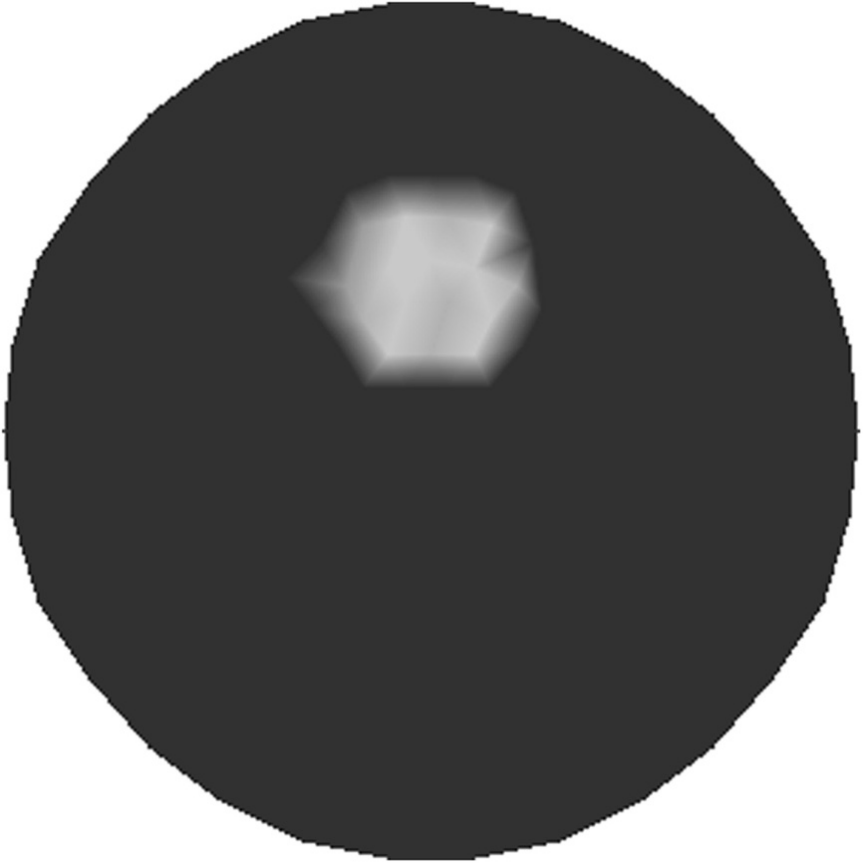
**Simulated phantom for tomographic reconstruction with a single target.** Single target with absorption coefficient *μ*_*axf*_ of 0.4 *mm* ^-1^, and the background medium with absorption coefficient *μ*_*axf*_ of 0.06 *mm* ^-1^.

Figure 2 displays the simulated phantom with a single fluorescent target. The phantom has eight sources and thirty detectors for the measurement.

**Figure 3:**
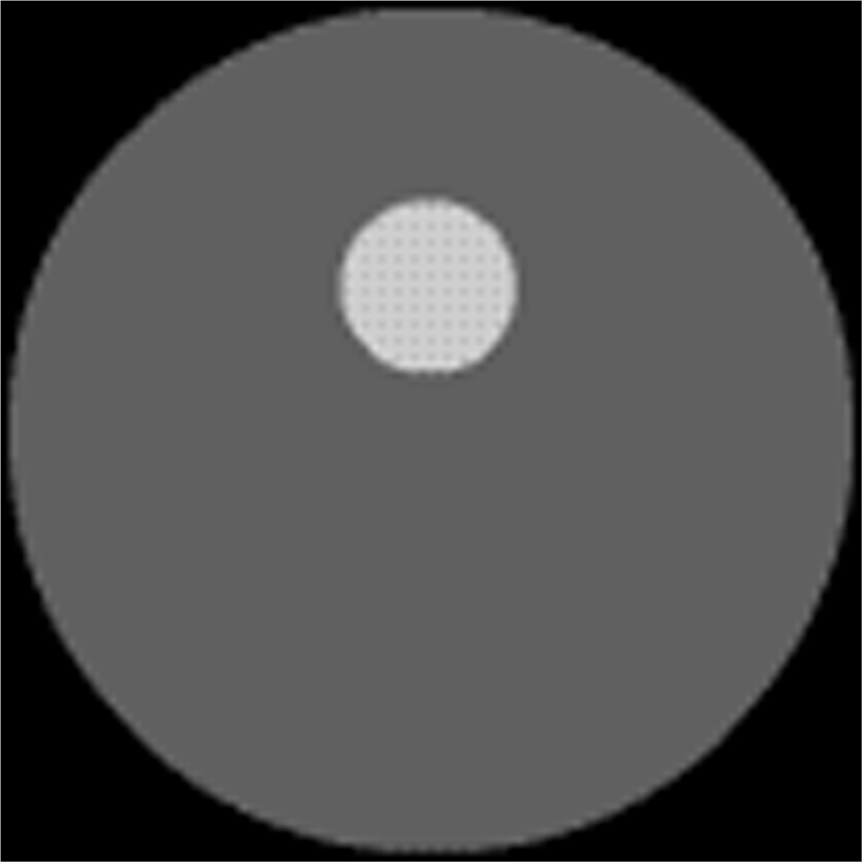
**Prior image used for one-target phantom.** The prior image is used to generate the adaptively refined mesh for reconstruction of one-target phantom.

Implementation of a finite-element simulation needs an appropriate mesh upon which to simulate the reconstruction results. In order to reduce the computational burden without greatly reducing the image resolution, the mesh used for reconstruction is refined adaptively using the *a priori* image displayed in Figure 3. First, the reconstruction domain is uniformly discretized. Then, the uniform mesh is refined for the areas with large variations of the pixel values in the *a priori* image. Whether the mesh needs to be refined is judged by following formula

**Figure 4:**
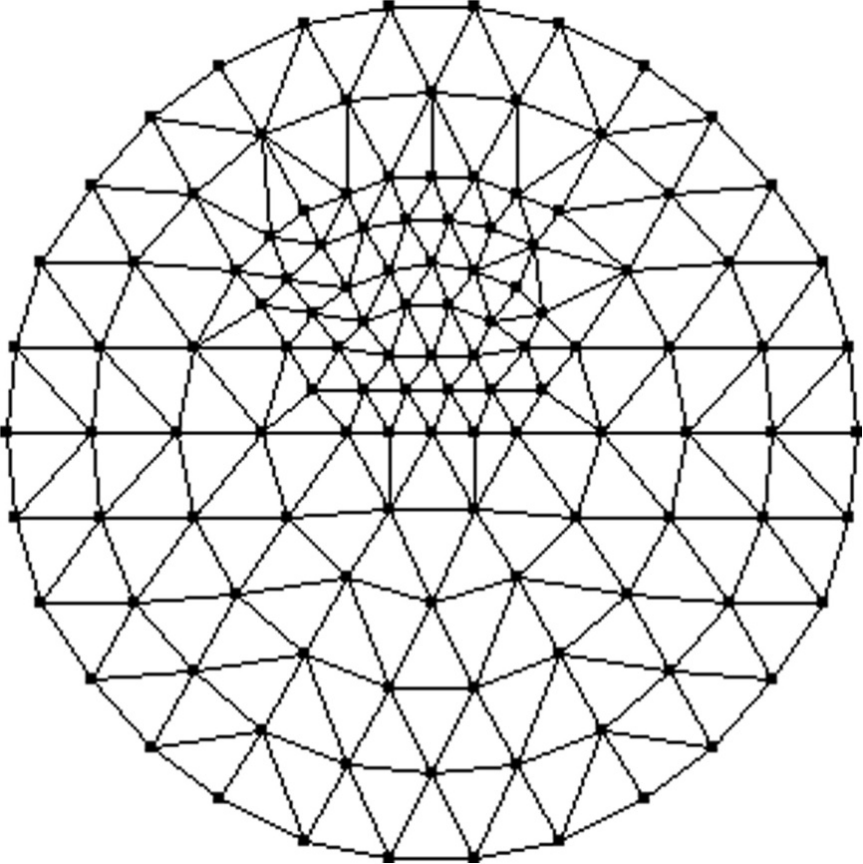
**Adaptively refined mesh of tomographic reconstruction for one-target phantom.** The adaptively refined mesh has 122 nodes and 212 elements.

19

where *X* denotes the pixel value in the prior image, *D* denotes the variation of pixel values in the triangle, and *E* denotes the expectation operator. The corresponding triangle will be refined when the variation is larger than the assumed threshold. In such a way, the adaptively refined mesh can be generated. As shown in Figure 4, the adaptively refined mesh has 122 nodes and 212 elements. The optical properties of the target and the background with regards to Figure 2 are given in Table 1.

**Table 1:** **Optical properties of one-target phantom**

Excitation light	***μ***_***axf***_(***mm*** ^- 1^)	***μ***_***axi***_(***mm*** ^- 1^)		ϕ	***τ***( ***ns***)
Target	0.4	0.04	5.0	0.3	0.8
Background	0.06	0.04	5.0	0.3	0.8
**Fluorescent light**	***μ***_***amf***_ **(** ***mm*** ^**- 1**^ **)**	***μ***_***ami***_ **(** ***mm*** ^**- 1**^ **)**		ϕ	***τ*** **(** ***ns*** **)**
Target	0.3	0.03	4.0	0.3	0.8
Background	0.006	0.03	4.0	0.3	0.8

**Figure 5:**
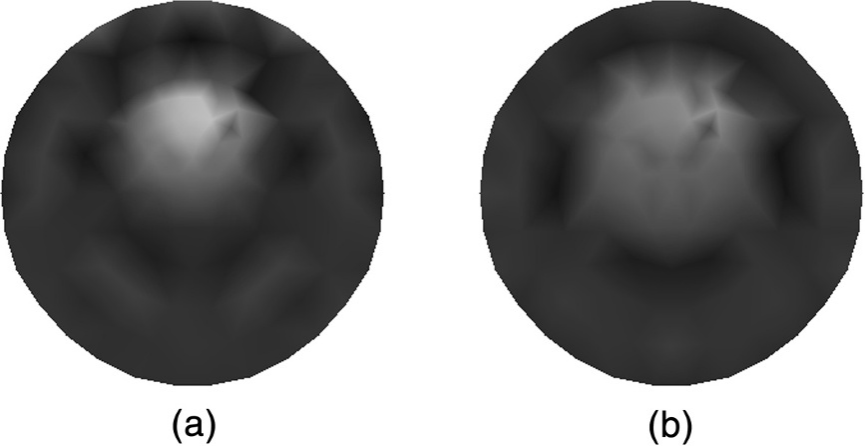
**Reconstruction results of absorption coefficient** ***μ***_***axf***_ **for one-target phantom. (a)** Reconstruction result with 30 measurements, and **(b)** reconstruction result with 18 measurements.

The reconstruction results of *μ*_*axf*_ for the single target case from the traditional method that without using CS are summarized in Figure 5. The tomographic reconstruction with 30 measurements is shown in Figure 5(a), and the reconstruction with 18 measurements is depicted in Figure 5(b). It is seen that the quality of image reconstruction for the location of target as well as the contrast can be improved with the increasing number of the measurements, which may lead to higher computational requirements.

**Figure 6:**
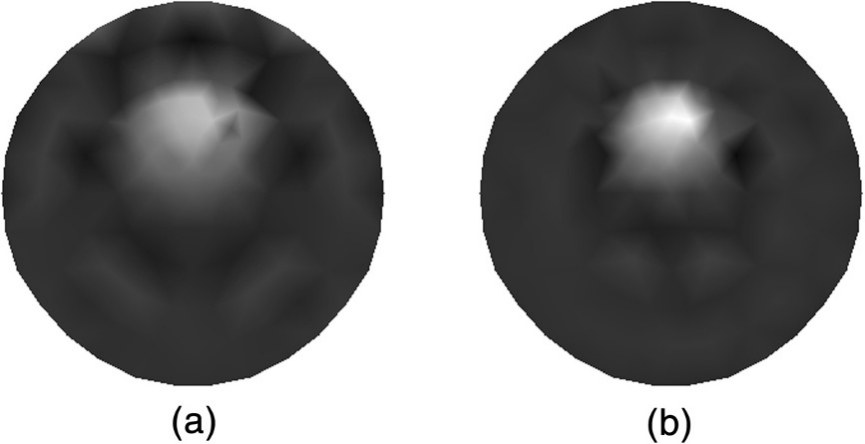
**Reconstruction results of absorption coefficient** ***μ***_***axf***_ **for phantom with a single target. (a)** Reconstruction result based on the traditional method, and **(b)** reconstruction result based on the proposed method.

The reconstructed absorption distributions for the single target case using the different algorithms are displayed in Figure 6, where Figure 6(a) shows the traditional reconstruction result with 30 measurements, and Figure 6(b) depicts the reconstruction result based on the proposed method with 15 measurements. As can be seen, the proposed algorithm can reconstruct the target with enhanced contrast and more accurate shape.

The accuracy of the reconstructions is analyzed quantitatively by computing the mean square error (MSE) from the distribution of the optical properties for the reconstructed data sets.
20

where *N* represents the total number of nodes in the domain. The superscript *cal* denotes the reconstructed results, and *act* denotes the actual distribution of the optical properties.

The quantitative performance of reconstruction for the one-target phantom in terms of the two metrics as computation time and MSE is given in Table 2 to further evaluate the reconstruction quality. Examining this table, we observe that our proposed algorithm can decrease the computation time of reconstruction as compared with the traditional method. Furthermore, Table 2 suggests that the proposed method can provide relatively high reconstruction quality with low computational requirements.

**Table 2:** **Performance comparison of reconstruction methods for phantom with one target**

Methods	Our algorithm	Traditional method
Computation time (s)	163	217
MSE	4.39 × 10^-4^	4.79 × 10^-4^

### Dual fluorescent targets

**Figure 7:**
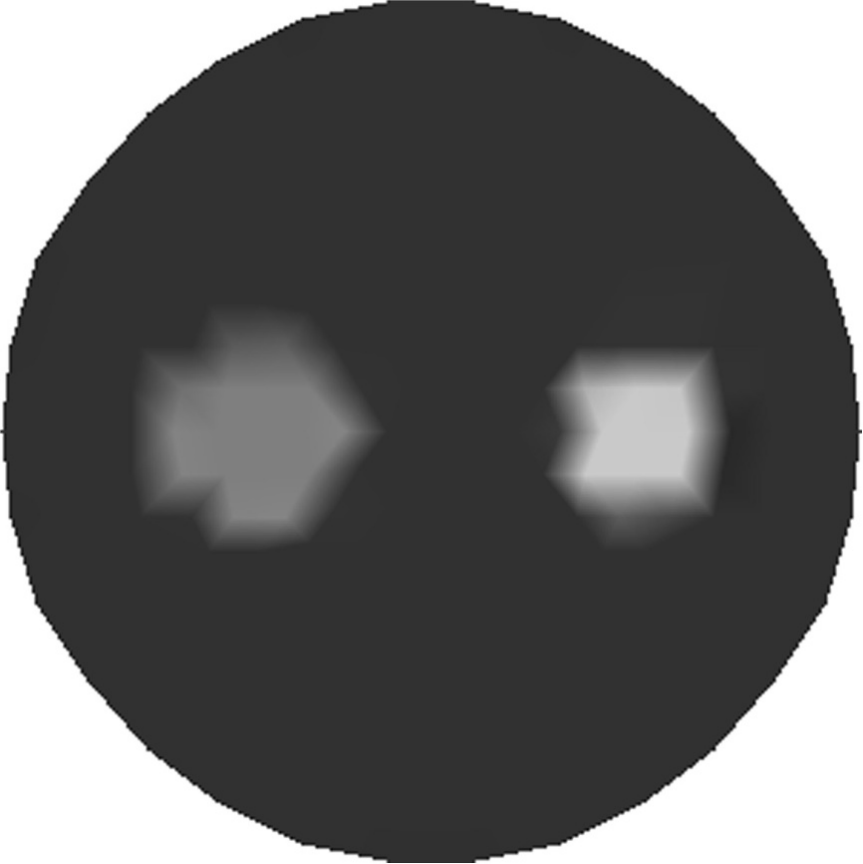
**Simulated phantom for tomographic reconstruction with dual targets.** One of the targets with low absorption coefficient *μ*_*axf*_ of 0.3 *mm* ^-1^, the other with high absorption coefficient *μ*_*axf*_ of 0.4 *mm* ^-1^, and the background medium with absorption coefficient *μ*_*axf*_ of 0.06 *mm* ^-1^.

The simulated phantom with dual fluorescent targets of different shapes is illustrated in Figure 7. The source–detector configurations of the two-target phantom are the same as the single target case.

**Figure 8:**
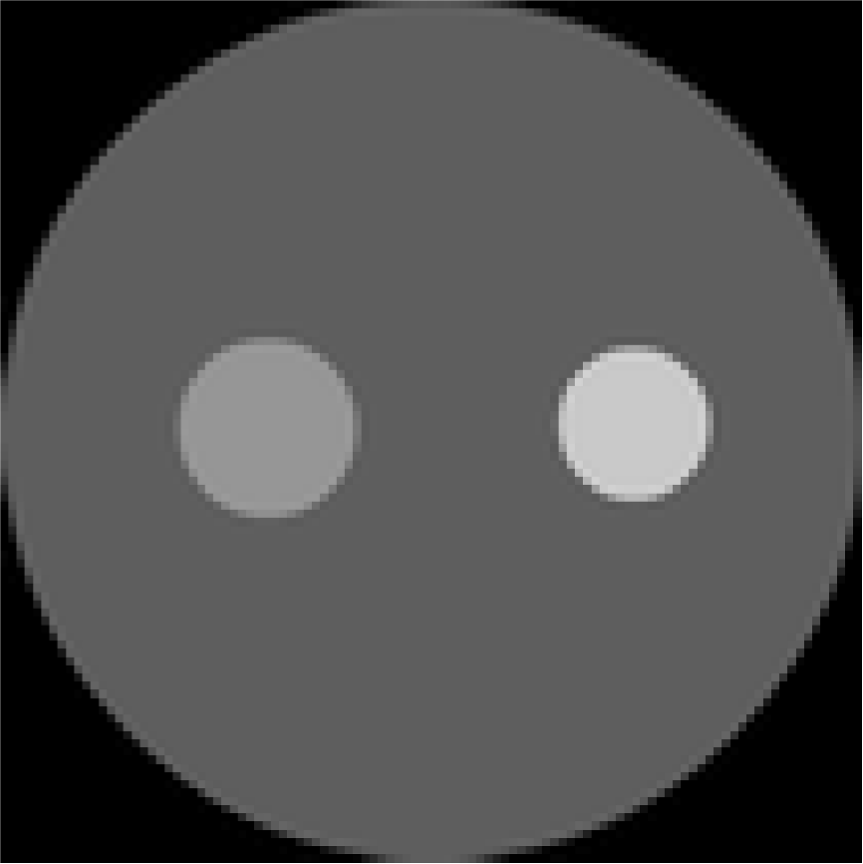
**Prior image used for two-target phantom.** The prior image is used to generate the adaptively refined mesh for reconstruction of two-target phantom.

**Figure 9:**
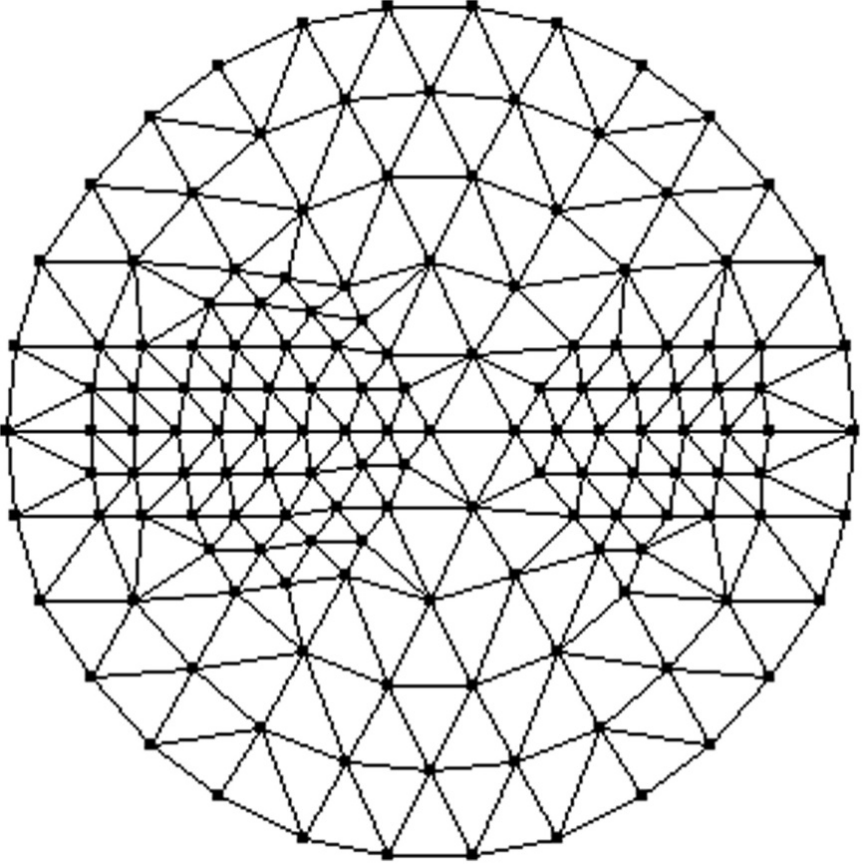
**Adaptively refined mesh of tomographic reconstruction for two-target phantom.** The adaptively refined mesh has 148 nodes and 264 elements.

Figure 8 depicts the prior image, which is introduced to refine the mesh for reconstruction. Thus, the adaptively refined mesh has 148 nodes and 264 elements as shown in Figure 9. Table 3 outlines the optical properties of the targets and the background with regards to Figure 7.

**Table 3:** **Optical properties of two-target phantom**

Excitation light	***μ***_***axf***_(***mm*** ^- 1^)	***μ***_***axi***_(***mm*** ^- 1^)	***μ*** ′ _***sx***_(***mm*** ^- 1^)	ϕ	***τ***( ***ns***)
Targets	0.3, 0.4	0.04	5.0	0.3	0.8
Background	0.06	0.04	5.0	0.3	0.8
**Fluorescent light**	***μ***_***amf***_ **(** ***mm*** ^**- 1**^ **)**	***μ***_***ami***_ **(** ***mm*** ^**- 1**^ **)**	***μ*** ′ _***sm***_ **(** ***mm*** ^**- 1**^ **)**	ϕ	***τ*** **(** ***ns*** **)**
Targets	0.03, 0.04	0.03	4.0	0.3	0.8
Background	0.004	0.03	4.0	0.3	0.8

**Figure 10:**
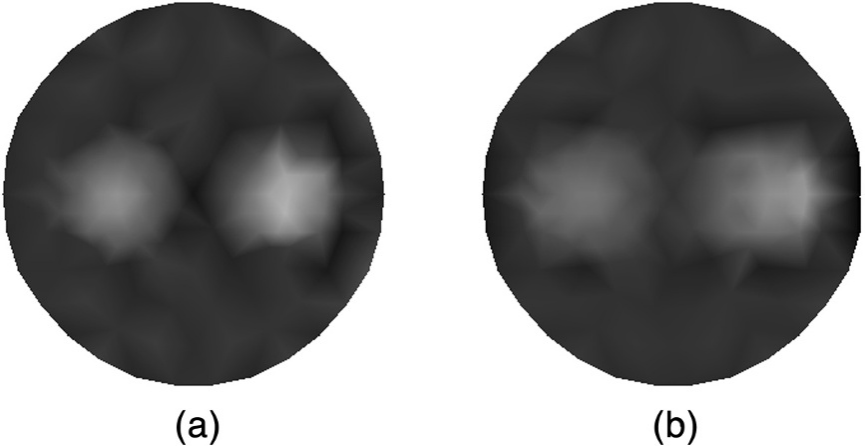
**Reconstruction results of absorption coefficient** ***μ***_***axf***_ **for two-target phantom. (a)** Reconstruction result with 30 measurements, and **(b)** reconstruction result with 18 measurements.

In Figure 10, the reconstructed images of *μ*_*axf*_ for the dual targets case with 30 measurements (see Figure 10(a)) and 18 measurements (see Figure 10(b)) are shown. The reconstructed results in Figure 10 are obtained based on the traditional method that without using CS. We can also observe that the higher accuracy of locations of the dual targets and the contrast from the reconstruction result can be achieved with the increasing measurements. On the other hand, the computational burden of reconstruction will become greater with more measurements.

**Figure 11:**
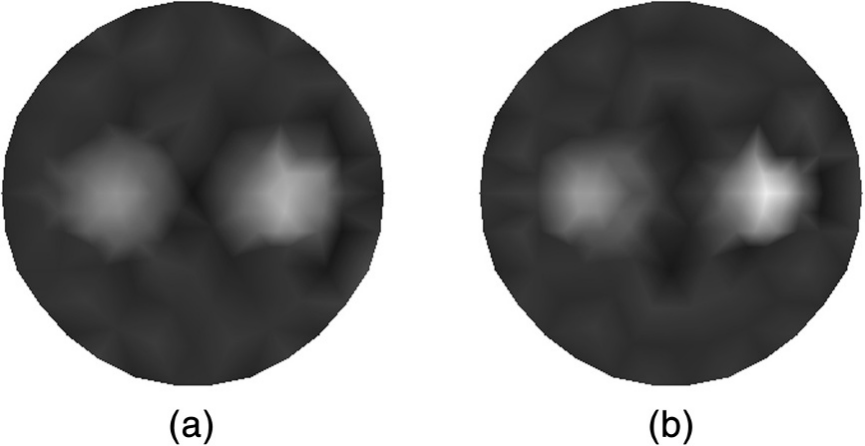
**Reconstruction results of absorption coefficient** ***μ***_***axf***_ **for phantom with dual targets. (a)** Reconstruction result based on the traditional method, and **(b)** reconstruction result based on the proposed method.

Figure 11 depict the reconstructed absorption distributions for the dual targets case using the different algorithms. The traditional reconstruction result with 30 measurements and that based on our method with 15 measurements are shown in Figure 11(a) and (b), respectively. We can observe that the shape and contour of the reconstructed targets with the proposed algorithm are better than those with the traditional method. Note also that the contrast can be improved with the proposed algorithm.

Table 4 lists the performance of reconstructions in terms of the computation time and MSE for the phantom with dual targets to assess the reconstruction quality. As can clearly be seen, the computation time and MSE of the reconstruction with our algorithm are less than those with the traditional method. In other words, for the dual targets case, our proposed algorithm can also improve the reconstruction speed with high accuracy.

**Table 4:** **Performance comparison of reconstruction methods for phantom with two targets**

Methods	Our algorithm	Traditional method
Computation time (s)	225	288
MSE	2.50 × 10^-4^	2.82 × 10^-4^

## Conclusions

In this work, we developed an innovative method based on CS for image reconstruction of FMT. A scheme of grouped sources is incorporated in the reconstruction process. In comparison to traditional reconstruction approach, the CS-based reconstruction algorithm has demonstrated significant advantages as faster speed and high accuracy. Furthermore, the cost and the amount of measurements for image reconstruction can be reduced with the CS-based reconstruction algorithm. This can be expected to have a significant impact on the clinical applications of FMT.

## Abbreviations

FMT: Fluorescence molecular tomography
CS: Compressed sensing
RTE: Radiative transfer equation
FEM: Finite element method
MSE: Mean square error.
